# Prevalence of workplace violence against health care workers in hospital and pre-hospital settings: An umbrella review of meta-analyses

**DOI:** 10.3389/fpubh.2022.895818

**Published:** 2022-08-08

**Authors:** Ali Sahebi, Mohamad Golitaleb, Siamak Moayedi, Mercedes Torres, Hojjat Sheikhbardsiri

**Affiliations:** ^1^Non-communicable Diseases Research Center, Ilam University of Medical Sciences, Ilam, Iran; ^2^Department of Nursing, School of Nursing, Arak University of Medical Sciences, Arak, Iran; ^3^Department of Emergency Medicine, University of Maryland School of Medicine, Baltimore, MD, United States; ^4^Health in Disasters and Emergencies Research Center, Institute for Futures Studies in Health, Kerman University of Medical Sciences, Kerman, Iran

**Keywords:** workplace violence, health care workers, occupational risk, health care professionals, violence

## Abstract

**Introduction:**

Workplace violence (WPV) is associated with adverse consequences for patients and health care workers (HCWs). The aim of this study was to assess the prevalence of WPV against HCWs in the hospital and pre-hospital settings.

**Methods:**

Using PRISMA guidelines, data resources including Scopus, PubMed, Web of Science, and Google Scholar were used for the search. The searches were conducted without any time limit until the end of December 2021. The random-effects model was used for this meta-analysis. *I*^2^ index was used to examine heterogeneity and the Egger test was used to examine publication bias.

**Results:**

Of the 255 studies identified, 14 studies entered the umbrella review. The overall prevalence was as follows: WPV (58.7%); physical violence (20.8%); verbal violence (66.8%); and sexual harassment (10.5%).

**Conclusion:**

The prevalence of WPV and its types against HCWs is relatively high. WPV is associated with physical, psychological, and occupational consequences. Measures should be taken to reduce the consequences of WPV.

## Introduction

Workplace violence (WPV) is a situation in which a person is harassed, threatened, or attacked at work (1). According to the World Health Organization (WHO), WPV includes physical and psychological violence (2). WPV is a major issue in healthcare settings in both hospital and pre-hospital settings (3). Unfortunately, WPV is on the rise in all health care settings (4). Many factors such as night work, high stress, lack of resources, disproportionate gender representation, and inadequate workplace security can lead to WPV (5).

Health care workers (HCWs) are at high risk for exposure to WPV (6, 7). According to studies, 50–88% of HCWs have been exposed to WPV (3). Depending on the type and setting of the health care environments, the rates can be higher. For example, up to 90% of emergency medicine HCWs report some degree of WPV (4). Similarly, 83% of pre-hospital emergency medical technicians experience WPV at least once a year (8).

WPV for HCWs may be associated with negative consequences such as low job satisfaction, change of profession, and work absenteeism (9, 10). Additionally, the experience of WPV is associated with decreased self-esteem, increased anxiety, and stress (11–13). Furthermore, WPV toward HCWs can lead to reduced quality of patient care (14).

Numerous systematic review and meta-analysis studies of WPV against HCWs have been conducted, but a comprehensive evaluation summarizing the results is lacking. This umbrella review serves as a data reference for policymakers in the field of health care. The aim was to assess the prevalence of WPV against HCWs in hospital and pre-hospital settings.

## Methods

This study was conducted based on the Preferred Reporting Items for Systematic Reviews and Meta-Analyses (PRISMA) guidelines (15). The protocol of this study was registered in the International Prospective Register of Systematic Reviews (PROSPERO) with code CRD42022296244.

### Search strategy

Data resources including Scopus, PubMed, Web of Science, and Google Scholar were used to identify the studies. Related keywords, search fields and operators were used to formulate search strategies. Initially, a search strategy was developed for the PubMed database, and then other search strategies were designed in accordance with the PubMed database. The searches were conducted in English without any time limit until the end of December 2021. Search strategies for the types of databases are listed in Table 1.

**Table 1:** Search strategies for types of databases.

**Database**	**Search strategy**
Pubmed	((“Workplace violence*”[tiab] OR aggression* OR “harassment*” OR bullying OR “workplace bullying” OR assault* OR abuse OR “physical abuse” OR violence OR “assaultive behavior”) AND (“health care provider*” OR “health personnel” OR “healthcare provider*” OR “health care worker*” OR “medical staff” OR “medical worker*” OR “healthcare professional*”) AND (“systematic review”[tiab]) AND (“meta-analysis”[tiab] OR “meta-analytic”))
Scopus	((TITLE - ABS (“workplace violence*”) OR ALL(aggression*) OR ALL(“harassment*”) OR ALL(bullying) OR ALL(“workplace bullying”) OR ALL(assault*) OR ALL(abuse) OR ALL(“physical abuse”) OR ALL (violence) OR ALL(“assaultive behavior”)) AND (ALL(“health care provider*”) OR ALL(“health personnel”) OR ALL(“healthcare provider*”) OR ALL(“health care worker*”) OR ALL(“medical staff”) OR ALL(“medical worker*”) OR ALL(“health care professional*”)) AND (TITLE-ABS (“systematic review”)) AND (TITLE-ABS(“meta-analysis”) OR ALL(“meta-analytic”)))
Web of Science	((TS= (“workplace violence*”) OR TS= (aggression*) OR TS= (“harassment*”) OR TS= (bullying) OR TS= (“workplace bullying”) OR TS= (assault*) OR TS= (abuse) OR TS= (“physical abuse”) OR TS= (violence) OR TS= (“assaultive behavior”)) AND (TS= (“health care provider*”) OR TS= (“health personnel”) OR TS= (“health care provider*”) OR TS= (“healthcare worker*”) OR TS= (“medical staff”) OR TS= (“medical worker*”) OR TS= (“health care professional*”)) AND (TI= (“systematic review”)) AND (TI= (“meta-analysis”) OR TS= (“meta-analytic”)))

### Eligible criteria

All studies that reported the prevalence of WPV and its types in HCWs by meta-analysis were included. Exclusion criteria were the prevalence of WPV in home health workers and non-meta-analysis studies.

### Selection of studies

EndNote X7 software was used to manage search results. The initial identified studies were entered into this software. Duplicates were removed and exclusion/inclusion criteria were applied. The full text of the remaining studies were independently reviewed by two researchers (AS, MG) and the final studies were selected for quality evaluation.

### Quality assessment and data extraction

Two researchers (AS, MG) independently used the AMSTAR-2 (A Measurement Tool to Assess Systematic Reviews, version 2) tool for qualitative evaluation of the selected studies (16). This tool has 16 items and the answer to each question is binary. The results were classified into four critical levels: Very low, low, medium, and high. For data extraction, the same two researchers, independently used a checklist designed in Word 2016 software to extract data including the first author of the study, place of study, number of reviewed studies, heterogeneity, publication bias, the prevalence of WPV and its variants from each study.

### Statistical analysis

The random-effects model was used for the meta-analysis. Heterogeneity between studies was assessed by the *I*^2^ index. Heterogeneity <25%, 25–50%, 50–75, and more than 75%, respectively, indicated no, moderate, high, or very high heterogeneity (17, 18). Publication bias was assessed using the Egger test. The data was analyzed using STATA software (version 14).

## Results

### Search results

**Figure 1:**
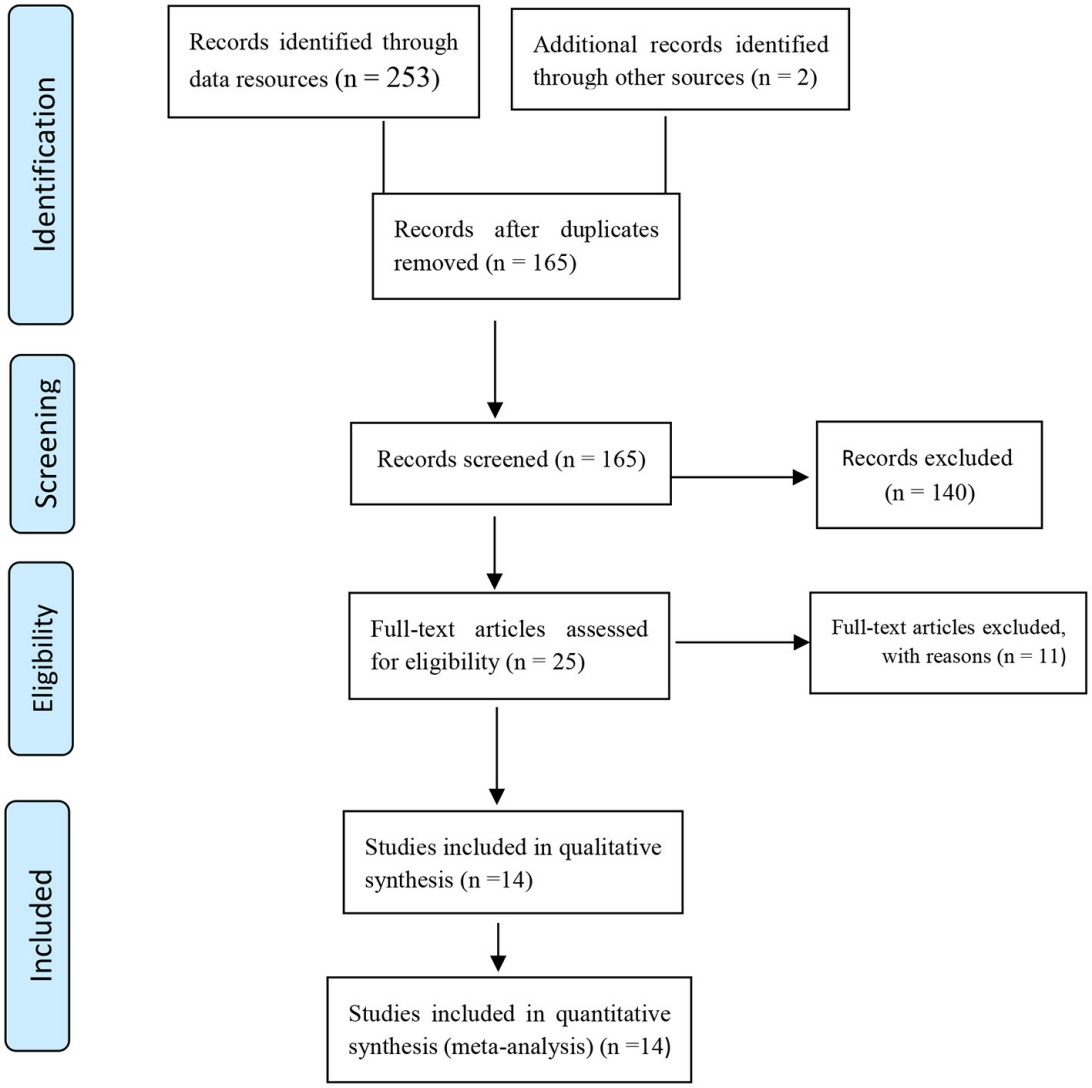
Flowchart of the selection of studies based on PRISMA.

After the comprehensive search, 255 initial studies were identified and after removing duplicates, 165 studies were screened and finally, 14 studies were qualitatively evaluated and entered the meta-analysis phase. Figure 1 shows the selection stages of the studies.

### Characteristics of studies

In the 14 final studies included in the meta-analysis, 674,266 health care workers were studied. Table 2 shows the characteristics and data of each study separately.

**Table 2:** The characteristic of studies included in the umbrella review and meta-analysis.

**References**	**Location**	**Sample size**	**Prevalence of overall WPV**	**Number of studies**	**Heterogeneity (*I*^2^)**	**Publication bias**	**Group studied**
Azami et al. (19)	Iran	10,858	Verbal: 80.8% (95% CI:74.2–86.0) Physical: 24.8% (95% CI: 17.4–34)	26	Physical (*I*^2^ = 97%) Verbal (*I*^2^ =97.76%)	Begg and Egger's tests (no publication bias)	Nurses
Sahebi et al. (20)	Iran	1,257	Physical: 36.39% (95%CI: 27.29–45.50) Verbal: 73.13% (95% CI: 68.64–77.62)	9	Physical (*I*^2^ = 90.8%) Verbal (*I*^2^ = 62.7%) Cultural (*I*^2^ = 94.7%)	Begg's (*p* = 0.361)	Emergency Medical Personnel
Li et al. (21)	China	61,800	Physical: 19.33% (95% CI: 16.49–22.53%)	65	*I*^2^ = 98.8%	Begg's (*P* = 0.1012)	HCWs
Varghese et al. (22)	India	42,222	WPV: 58% (95% CI: 51–64) Verbal: 64% (95%CI: 59–70) Physical: 23% (CI: 14–34) Sexual harassment: 12% (95%CI: 7–17)	38	WPV (*I*^2^ = 99.26%) Verbal (*I*^2^ = 98.78%) Physical (*I*^2^ = 99.68%)	Egger's WPV (*P* = 0.18) Verbal (*p* = 0.81) Physical (*p* = 0.561)	Nurses
Nowrouzi-Kia et al. (23)	Canada	10,786	WPV: 69% (95% CI: 58–78)	6	*I*^2^ = 0.974	-	Physicians
Hossain et al. (24)	India	2,849	Verbal: 52% (95% CI: 45–60) Physical: 8% (95% CI: 5–11) WPV: 63% (95% CI: 54–72)	15	WPV (*I*^2^ =96.15%) Verbal (*I*^2^ = 93.90%) Physical (*I*^2^ = 93.70%)	-	HCWs
Dalvand et al. (25)	Iran	5,639	Verbal: 74% (95% CI: 66–83) Physical: 28% (95% CI: 21–35)	22	-	Egger's (*P* = 0.03)	Nurses
Liu et al. (26)	China	331,544	Physical: 24.4% (95% CI 22.4–26.4) Verbal: 57.6% (95% CI 51.8–63.4) Sexual harassment: 12.4% (95% CI: 10.6–14.2) WPV: 61.9% (95% CI 56.1–67.6)	158	-	-	HCWs
Liu et al. (27)	China	22,968	WPV: 71% (95% CI: 67–75) Verbal: 63% (95% CI: 58–67). Physical: 14% (95% CI: 11–18) Sexual harassment was 6% (95%CI: 4–9)	38	WPV (*I*^2^ = 98%) Physical (*I*^2^ = 98%) Verbal (*I*^2^ = 98%)	Egger's (no publication bias)	Nurses
Lu et al. (28)	China	78,026	WPV: 62.4% (95% CI: 59.4–65.5) Physical: 13.7% (95% CI:12.2–15.1) Verbal: 61.2% (95% CI: 55.1–67.4) Sexual harassment: 6.3% (95% CI: 5.3–7.4)	44	Physical (*I*^2^ = 97.1) Verbal (*I*^2^ = 99%) Sexual harassment (*I*^2^ = 96.5%)	*WPV:* Begg's *(p* = 0.229)	HCWs
Shabanikiya et al. (29)	Iran	8,694	WPV: 66 % (95% CI: 20–111) Physical: 25% (95%CI: 16–34), Verbal: 58% (95%CI: 29–86) Sexual harassment: 16% (95%CI: 9–22)	11	WPV (*I*^2^ = 99.94%) Physical (*I*^2^ = 99.31%)	*Egger's (P = 0.094)*	Emergency Medical Personnel
Zeng et al. (30)	China	39,486	sexual harassment: 7.5% (95% CI: 5.5–10.1)	37	*I*^2^ = 97.79%	*Egger's (P = 0.57)*	Nurses
Lu et al. (31)	China	52,345	Sexual harassment in last 12 month: 13% (11–14) Sexual harassment in nursing career: 53.4% (95% CI: 23.1–83.7)	34	Sexual harassment in last 12 month (*I*^2^ = 98.6%) Sexual harassment in Nursing career (*I*^2^ = 99.7%)	*-*	Nurses
Aljohani et al. (32)	USA	5,792	Verbal: 77 % (95% CI: 72–82) WPV: 24 % (95% CI: 18–31)	22	Verbal (*I*^2^ = 97%) WPV (*I*^2^ = 93%)	*-*	HCWs

*HCWs, Healthcare Workers*.

### Meta-analysis results

**Figure 2:**
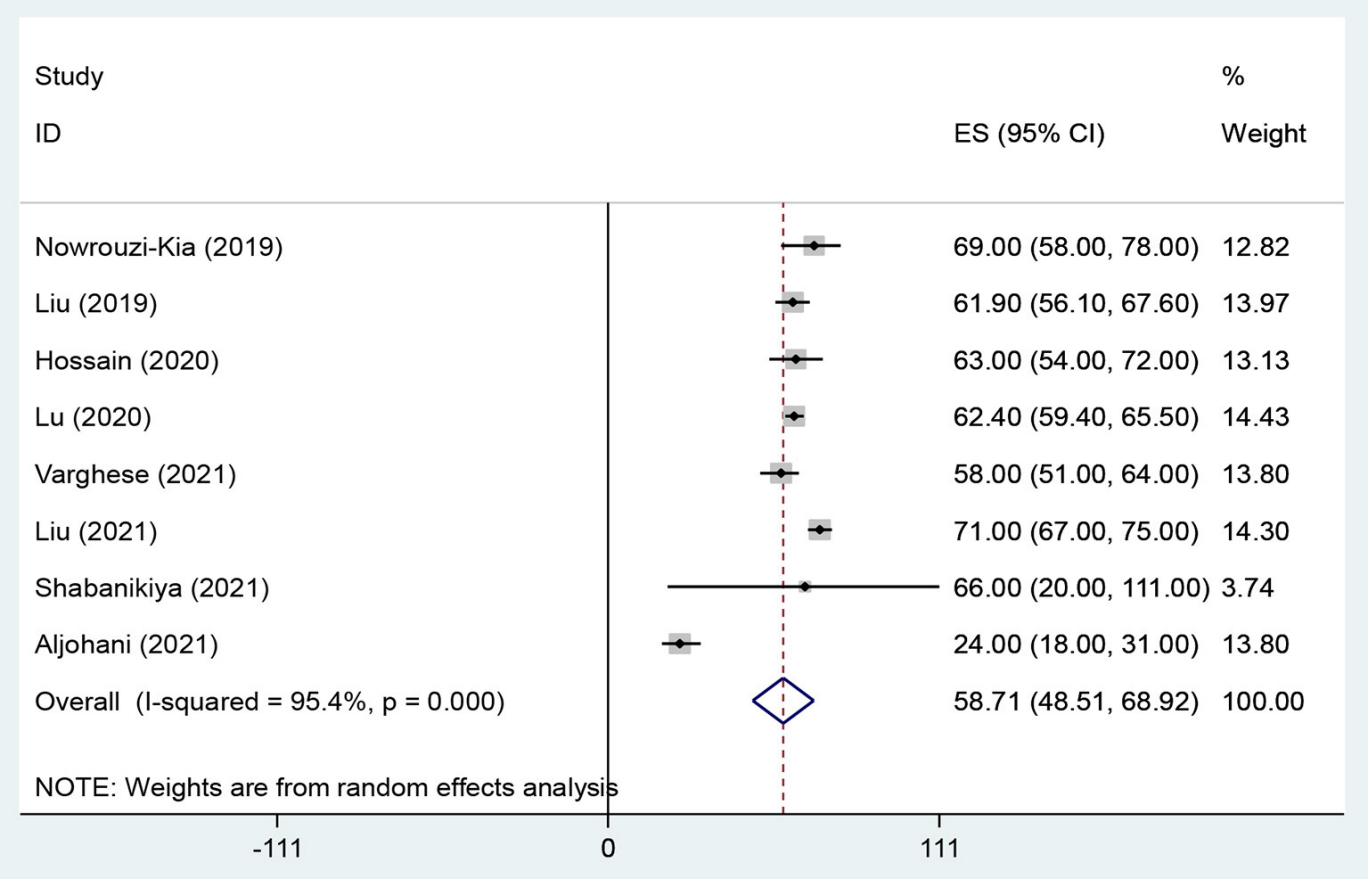
The Forest plot of overall and individual prevalence of WPV with 95% confidence interval.

**Figure 3:**
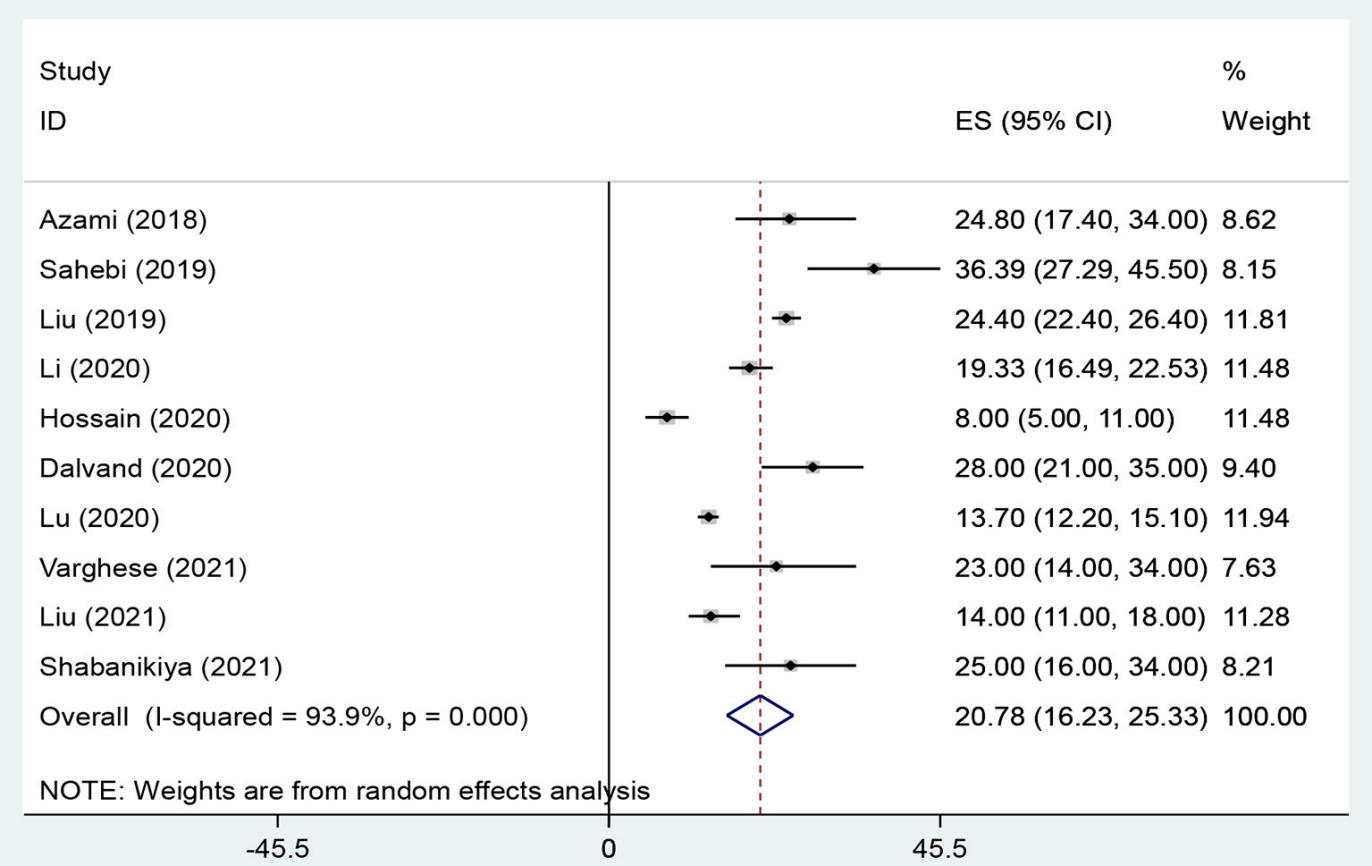
The Forest plot of overall and individual prevalence of physical violence with 95% confidence interval.

**Figure 4:**
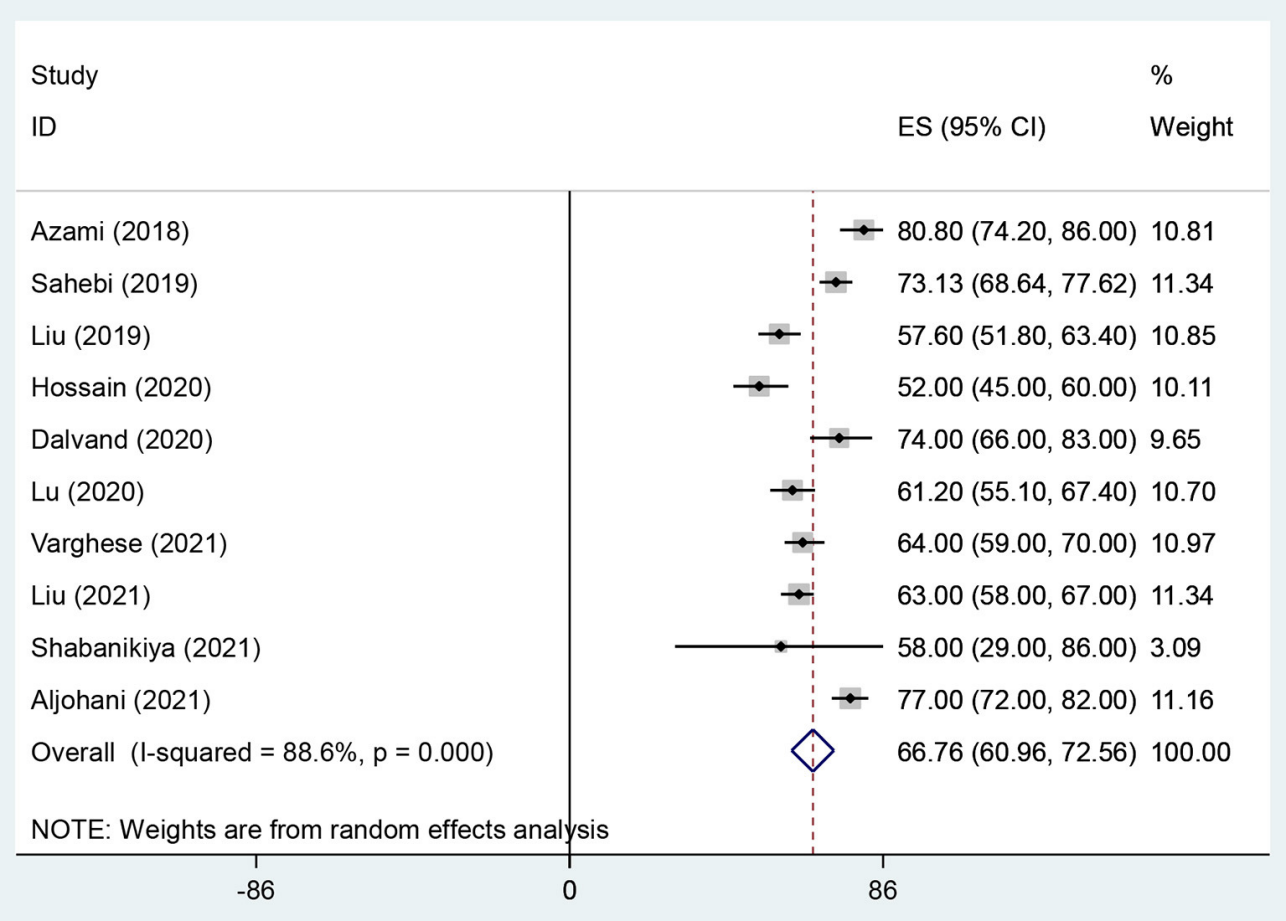
The Forest plot of overall and individual prevalence of verbal violence with 95% confidence interval.

**Figure 5:**
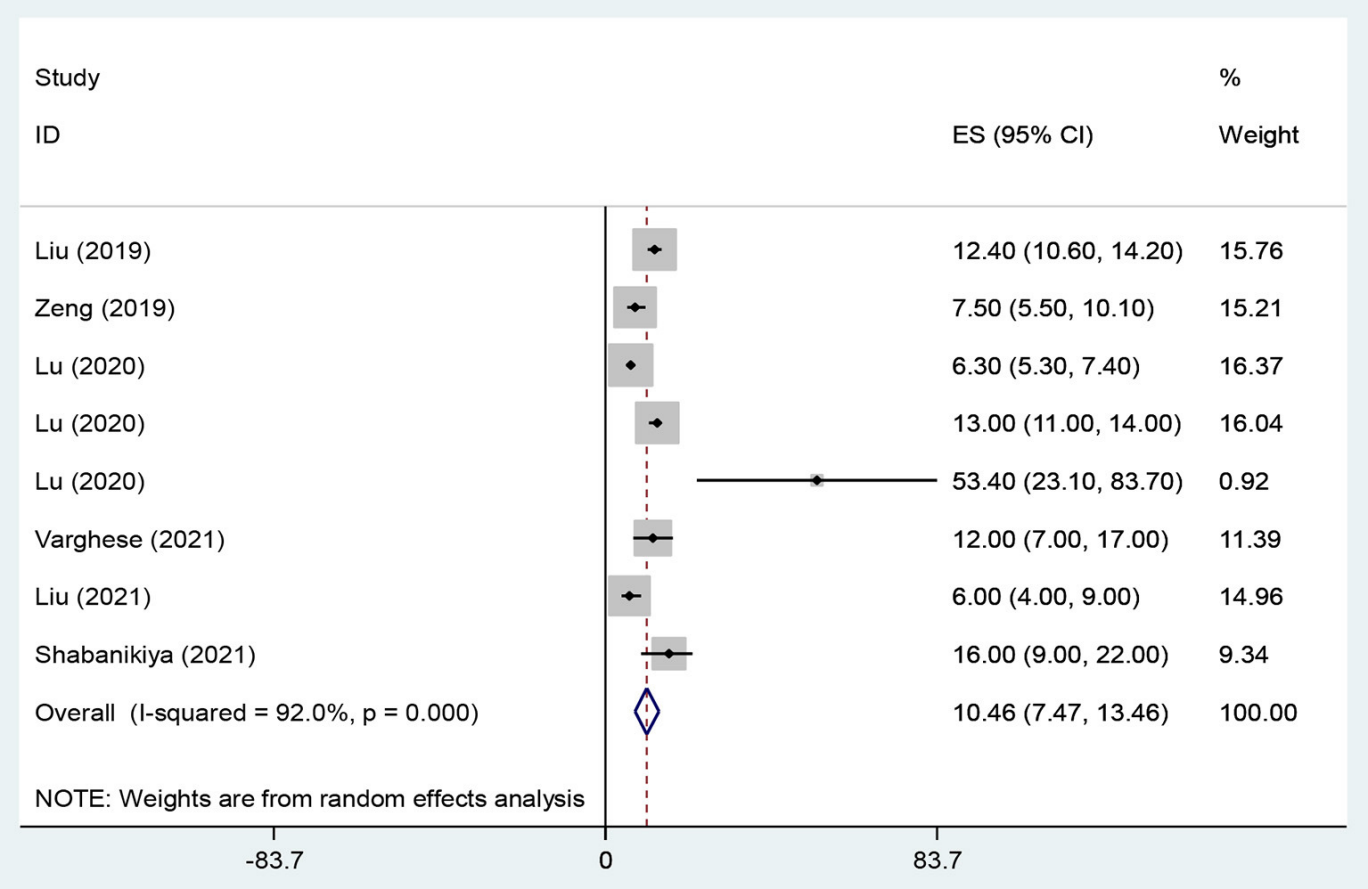
The Forest plot of overall and individual prevalence of sexual harassment with 95% confidence interval.

The overall prevalence of WPV, physical violence, verbal violence and sexual harassment in health care workers were 58.7% (95% CI: 48.51–68.92, *I*^2^ = 95.4%, *p* < 0.001) (Figure 2), 20.8% (95% CI: 16.23–25.33, *I*^2^ = 93.9%, *p* < 0.001) (Figure 3), 66.8% (95% CI: 60.96–72.56, *I*^2^ = 88.6%, *p* < 0.001) (Figure 4), and 10.5% (95% CI: 7.47–13.46, *I*^2^ = 92%, *p* < 0.001) (Figure 5) respectively. The *I*^2^ index showed that the heterogeneity between the studies is very high. Based on Egger's test, publication bias in overall of WPV (*P* = 0.629), physical violence (*P* = 0.256), verbal violence (*P* = 0.600), and sexual harassment (*P* = 0.263) was not significant.

## Discussion

The prevalence of overall WPV, physical violence, verbal violence, and sexual harassment against HCWs are 58.7, 20.8, 66.8, and 10.5%, respectively. A 2020 meta-analysis study by Byon et al. showed that the prevalence of physical and non-physical violence against home health workers was 36.4 and 41.8%, respectively (33). Another meta-analysis showed that the prevalence of sexual violence among home health workers is 6% (34). We surmise that critical medical conditions, delirium, personality disorders, and lack of long-term relationships in hospital and pre-hospital settings lead to this higher prevalence of violence against HCWs than home health workers. Negative factors such as lack of information, inadequate personnel, and equipment increase the risk of WPV in health care services (35). Furthermore, in health care settings, factors such as work stress, poor co-worker relationships, and poor social support can lead to WPV (36).

The prevalence of violence, especially verbal violence against HCWs in hospital and pre-hospital settings is high. Sun et al. showed that the prevalence of verbal WPV against doctors in China was 76.2% (37). Magnavita et al. showed that WPV has a direct relationship with sleep problems (38). Other review studies have shown that WPV reduces the quality of work, increases mental health problems, and can lead to HCWs quitting their jobs (39). Additionally, workplace violence is directly related to burnout, lower job satisfaction, less patient safety, and an increase in medical mistakes (40–42). Therefore, health care policymakers should constantly screen for WPV and establish regulations and enact programs to minimize its occurrence.

## Conclusion

The prevalence of WPV against HCWs is high. WPV has negative physical, psychological, and occupational consequences for HCWs and their patients. Therefore, the health system managers should screen HCWs for the occurrence and impacts of WPV and establish regulations to minimize them.

## Limitations

In most of the included studies, the prevalence of WPV in men and women was not reported separately, so it was not possible to report the prevalence of WPV by gender. Another limitation of this study was the high degree of heterogeneity between the studies. Finally, the studies selected were in the English language and primarily from Asian continent sources. Thus, the results may not be generalizable to worldwide communities.

## Implication of this research

The authors used a systematic review and meta-analysis approach in this study for two reasons. First, an umbrella review of meta-analyses provides a higher level of evidence than other studies that review and evaluate original studies. Second, this is the first study in this field with this title. Numerous systematic reviews and meta-analyses have been conducted on the mental health outcomes, among health care workers **(**HCWs). Still, no single study has combined these results to identify overarching trends or conclusions. Therefore, the present umbrella review of meta-analyses aims to serve as the first and most comprehensive study in this regard. This umbrella review assesses all meta-analyses conducted on the prevalence of workplace violence against health care workers in hospital and pre-hospital settings among HCWs worldwide to estimate the prevalence of workplace violence against in this population. This study's results can serve as a resource for policy-makers or health managers to implement appropriate plans to improve the mental health of HCWs around the world.

## Data availability statement

The datasets presented in this study can be found in online repositories. The names of the repository/repositories and accession number(s) can be found in the article/supplementary material.

## Ethics statement

This article was approved by the Ethical Committee of Karmen University of Medical Sciences with Reg. No. 401000012. The Ethic Approval Code is IR.KMU.REC.1401.074.

## Author contributions

AS, MG, SM, MT, and HS designed the review, developed the inclusion criteria, screened titles and abstracts, appraised the quality of included papers, and drafted the manuscript, reviewed the study protocol and inclusion criteria and provided substantial input to the manuscript, and reviewed the study protocol. AS and MG read and screened articles for inclusion. All authors critically reviewed drafts and approved the final manuscript.

## Conflict of interest

The authors declare that the research was conducted in the absence of any commercial or financial relationships that could be construed as a potential conflict of interest.

## Publisher's note

All claims expressed in this article are solely those of the authors and do not necessarily represent those of their affiliated organizations, or those of the publisher, the editors and the reviewers. Any product that may be evaluated in this article, or claim that may be made by its manufacturer, is not guaranteed or endorsed by the publisher.
